# COVID-19 Is a Multi-Organ Aggressor: Epigenetic and Clinical Marks

**DOI:** 10.3389/fimmu.2021.752380

**Published:** 2021-10-08

**Authors:** Mankgopo Magdeline Kgatle, Ismaheel Opeyemi Lawal, Gabriel Mashabela, Tebatso Moshoeu Gillian Boshomane, Palesa Caroline Koatale, Phetole Walter Mahasha, Honest Ndlovu, Mariza Vorster, Hosana Gomes Rodrigues, Jan Rijn Zeevaart, Siamon Gordon, Pedro Moura-Alves, Mike Machaba Sathekge

**Affiliations:** ^1^ Nuclear Medicine Research Infrastructure (NuMeRI), Steve Biko Academic Hospital, Pretoria, South Africa; ^2^ Department of Nuclear Medicine, University of Pretoria & Steve Biko Academic Hospital, Pretoria, South Africa; ^3^ Department of Nuclear Medicine, Steve Biko Academic Hospital, Pretoria, South Africa; ^4^ SAMRC/NHLS/UCT Molecular Mycobacteriology Research Unit, DSI/NRF Centre of Excellence for Biomedical TB Research, Department of Pathology and Institute of Infectious Disease and Molecular Medicine, Faculty of Health Sciences, University of Cape Town, Cape Town, South Africa; ^5^ Nuclear and Oncology Division, AXIM Medical (Pty), Midrand; ^6^ Precision Medicine and SAMRC Genomic Centre, Grants, Innovation, and Product Development (GIPD) Unit, South African Medical Research Council, Pretoria, South Africa; ^7^ Laboratory of Nutrients and Tissue Repair, School of Applied Sciences, University of Campinas, Campinas, Brazil; ^8^ South African Nuclear Energy Corporation, Radiochemistry and NuMeRI PreClinical Imaging Facility, Mahikeng, South Africa; ^9^ Graduate Institute of Biomedical Sciences, College of Medicine, Chang Gung University, Taoyuan City, Taiwan; ^10^ Sir William Dunn School of Pathology, University of Oxford, Oxford, United Kingdom; ^11^ Ludwig Institute for Cancer Research, Nuffield Department of Medicine, University of Oxford, Oxford, United Kingdom

**Keywords:** ACE2, COVID-19, cytokine storm, epigenetics, multi-organ, pro-inflammatory cytokines, SARS-CoV-2, TMPRSS2

## Abstract

The progression of coronavirus disease 2019 (COVID-19), resulting from a severe acute respiratory syndrome coronavirus 2 (SARS-CoV-2) infection, may be influenced by both genetic and environmental factors. Several viruses hijack the host genome machinery for their own advantage and survival, and similar phenomena might occur upon SARS-CoV-2 infection. Severe cases of COVID-19 may be driven by metabolic and epigenetic driven mechanisms, including DNA methylation and histone/chromatin alterations. These epigenetic phenomena may respond to enhanced viral replication and mediate persistent long-term infection and clinical phenotypes associated with severe COVID-19 cases and fatalities. Understanding the epigenetic events involved, and their clinical significance, may provide novel insights valuable for the therapeutic control and management of the COVID-19 pandemic. This review highlights different epigenetic marks potentially associated with COVID-19 development, clinical manifestation, and progression.

## Main Background

**Figure 1:**
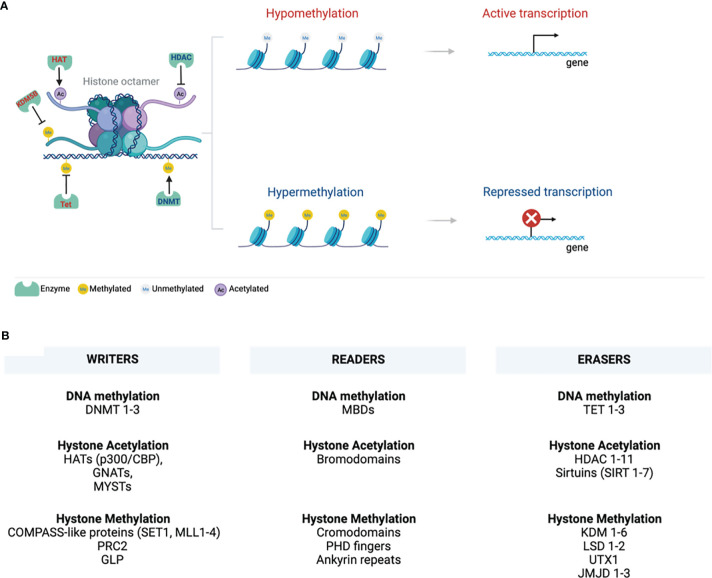
Chromatin structure. **(A)** A 147bp DNA wraps around the histone octamer with two copies of each of the histones H2A, H2B, H3, and H4. Various epigenetic mechanisms that modify chromatin, such as DNA methylation and histone modifications, are highlighted. DNA and histone methylation collaborate with different modifying enzymes and creates a tightly packed chromatin and suppress gene transcription by preventing the transcription machinery from binding DNA. Histone acetylation perturbs structural electrostatic interactions between the DNA and histones, resulting in the less compact structure of chromatin structure. This allows DNA access by transcription factors that promote gene transcription. **(B)** Writing, erasing, and reading chromatin methylation markers are highlighted. These mark various sites on the tail and globular domains of histones. Writers and erasers are methyltransferases and demethylases, respectively. These are recognised by distinct effector proteins called readers. (Created with BioRender.com) ac, Acetylation; DNMT, DNA methyltransferase; GLP, G9a-like protein; GNATs, Gcn5-related N-acetyltransferases; HATs, Histone acetyltransferases; HDACs, Histone deacetylases; JmjC, Jumonji C; KDM, Histone lysine demethylases; LSD, Lysine-specific demethylases; MBDs, Methyl-CpG binding domains; me, Methylation; MLL, Mixed-lineage leukaemia; PHD - Plant homeodomain; PRC2, Polycomb repressive complex 2; p300/CBP, p300 and cyclic AMP response element-binding protein; SET1, Suppressor of variegation 3–9, Enhancer of Zeste, Trithorax 1; SIRT, sirtuins; TET, Ten-eleven translocation; UTX1, Ubiquitously transcribed tetratricopeptide repeat, X chromosome 1.

Epigenetics is a branch of biology arising from inheritable gene transcription alterations in response to environmental cues, such as pollutants, chemicals, radiation, diet, stress, and pathogenic organisms (1). Epigenetic phenomena do not cause any genetic alterations or mutations. However, as the new phenotypes that are somatically heritable, epigenetic tags alter gene transcription and normal functions. Epigenetic marks are either suppressive or active and include DNA methylation, histone modification/chromatin remodelling, non-coding RNA, and RNA modification (**Figure 1**). These marks are implicated in activating or suppressing gene promoters, bodies, or transposable elements in normal processes such as ageing, genomic imprinting, and X-chromosome inactivation (2). DNA methylation is the best-studied stable epigenetic mark that occurs within CpG island promoter regions enriched with >70% of CpG (cytosine phosphate guanine) sites in the genome (3). It involves tagging or deposition of the methyl group of 5-methylcytosine to the DNA molecule through catalysis by DNA methyltransferases (DNMTs), which can be reversed by another family of enzymes called ten-eleven translocation (Tet 1-3) methyldioxygenases (4). DNMTs are regarded as writers of DNA methylation, recognised or read by methyl-CpG binding domains (MBDs) and then erased by TETs (**Figure 1**).

Eukaryotic cell DNA is packaged into chromatin wrapped around an octamer of four core histone proteins (5). Histones can be post-translationally modified by repressive or active histone marks that impact the interaction of histones with DNA or the occupancy of transcriptional machineries for gene expression (**Figure 1**). They dictate the chromatin transcriptional state of the local genomic regions *via* histone methylation, acetylation, ubiquitination, and phosphorylation. Chromatin forms a higher-order structure classified as euchromatin and heterochromatin (6). Euchromatin is a loosely packed or open form of chromatin enriched with DNA accessible to regulatory transcription complexes and promotes active gene transcription. Excessive acetylation of histone lysine residues is a common feature of euchromatin (7). It correlates with COMPASS-like proteins as binding partners and methylation of lysine 4 of histone 3 (H3K4), H3K36, and H3K79 that mark transcriptional activation of enhancers, gene promoters, and transcribed genes in gene bodies, respectively (8–10). Lysine can be mono-(me1), di- (me2), or tri-methylation (me3), providing unique functionality to each methylation site (9, 11). A tight or closed form of chromatin is called heterochromatin, protecting the DNA from being accessible to repressive transcriptional marks that restrict gene expression. Heterochromatin is further categorized into constitutive and facultative heterochromatins that are enriched in hypoacetylated or hypomethylated histones (9). The former is a stable form of heterochromatin comprised of repetitive DNA sequences (called DNA satellites) located at the transposon elements, centromere, and telomere. It is characterised by a repressive H3K9 epigenetic mark and heterochromatin protein 1 (HP1) chromodomain binding partner (8, 9, 12–14). Facultative heterochromatin is enriched with long interspersed nucleotide elements (LINE)-type sequences, repressive H3K27me2/3 epigenetic mark and its binding partner, polycomb repressive complex 2 (PRC2)-enhancer of zeste homolog 2 (EHZ2) (15, 16).

Writers, readers, and erasures of DNA methylation and histone modifications are listed in **Figure 1**. This review will discuss the role of epigenetics in COVID-19 infection, susceptibility to infection, and clinical markers established systemically during COVID-19 and may be associated with various epigenetic alterations.

## Mechanisms Of SARS-COV-2 Viral Infection And Multi-Organ System Invasion

### ACE2 and TMPRSS2: Viral Entry and Regulation

Severe acute respiratory syndrome coronavirus 2 (SARS-CoV-2) is the aetiological agent of the current pandemic, coronavirus disease 2019 (COVID-19) (17). This pathogen is enabled by the angiotensin-converting enzyme 2 (ACE2) (18). Mechanistically, SARS-CoV-2 penetrates and enters the host cell by binding to the ACE2 receptor as the primary target. This is facilitated by proteolytic priming by the cellular transmembrane serine protease 2 (TMPRSS2) (19). In the proposed model of respiratory failure, SARS-CoV-2 downregulates ACE2 through the SARS-CoV spike (SARS-S) protein, explaining the renin-angiotensin-aldosterone systems (RAAS) dysregulation and cardiotoxicity in severe COVID-19 infection (20). Suppression of ACE2 also induces tumour necrosis factor alpha (TNF-α) converting enzyme (TACE) that antagonises ACE2 shedding of the SARS-S (19). Modulation of TACE activity by SARS-S protein was found to depend on the cytoplasmic domain of ACE2 as ACE2 mutants devoid of the carboxyl-terminal region could not induce ACE2 shedding or TNF-α production (21). Moreover, deletion of the cytoplasmic tail of ACE2 or knock-down of TACE expression significantly attenuates viral infection (21). It has been shown that Ang II induces ACE2 shedding by promoting TACE activity as a positive feedback mechanism, suggesting that SARS-CoV mediated ACE2 down-regulation will promote Ang II accumulation and HIF-1α activation, which positively activates disintegrin and metalloproteinase domain-containing protein 17 (ADAM17) activity, thus perpetuating membrane shedding of ACE2, RAAS overactivation, and inflammation (22–26). This mechanism, however, is not universal to all coronaviruses because the spike protein of HNL63-CoV (NL63-S), a coronavirus that also utilizes ACE2 and is known to cause common influenza, did not produce similar cellular responses (21).

#### Lung as the Primary Target for SARS-CoV-2 Infection

SARS-CoV-2 infection is primarily a respiratory infection that targets type II alveolar epithelial cells (83%) in the lungs (27, 28). Upregulation of ACE2 in various cells usually disrupts ACE2 normal function from cleaving and converting angiotensin II to angiotensin 1-7 for tissue protection (29). SARS-CoV-2-infected type II alveolar epithelial cells leads to inflammation and severe damage in the lung tissue that is clinically manifested by elevated levels of ferritin and D-dimer, and association with oxygen desaturation, chest pain, and disease progression as indicated by computed tomography (CT) pulmonary angiography (30, 31). Elevated levels of macrophage/monocyte colony-stimulating factor (M-CSF, also known as colony-stimulating factor 1 receptor), granulocyte-monocyte colony-stimulating factor (GM-CSF), and interleukin (IL)-6 have also been reported in the later stages of COVID-19 (32–34). This correlates with pneumonia and acute respiratory distress syndrome (ARDS) that may lead to organ failure as observed in severe or critical cases of COVID-19 (31, 33, 35–37).

Most recently, the study of Ferreira-Gomes et al. (38) has shown that cells isolated from bronchoalveolar lavage of intensive care unit (ICU) patients with severe COVID-19 cases were enriched with tumour growth factor-beta 1 (TGF-β1)-expressing Th17, regulatory T cells, and CD14-positive cells, immune cells that are usually recruited to fight the infection. TGF-β1 is a master regulator of immune reaction and pulmonary fibrosis in COVID-19 patients (39). Its expression was associated with SARS-CoV-2 spike protein-specific IgM, IgG (IgG1 and IgG2), and IgA (IgA1 and IgA2) antibodies that protect systemic organs and mucosal surfaces, respectively (38, 40). SARS-CoV-2 spike protein-specific antibodies were also an indication of ongoing immune reaction and damage in secondary organs from the spread of viral infection (41). In the early days of ICU admission, IgG antibodies are predominantly generated by IL-10/21 specific to SARS-CoV-2 proteins (42). As a result of clonal expansion, later these antibodies become somatically mutated, virus non-specific, and undergo switching as instructed by TGF-β1 (38, 43). Ferreira-Gomes and co-authors have demonstrated that TGF-β1 induces chronic immune reaction by regulating antibody switching from IgG to IgA and this correlates with prolonged ICU stays of more than seven days (38).

Overall, systemic COVID-19 infection is characterised by various immunoregulatory and pro-inflammatory cytokines such as IL-1β, IL-2, IL-6, IL-7, IL-10, IL-18, D-Dimer, C-reactive protein (CRP), GM-CSF, interferon gamma-induced protein 10 (IP10), macrophage inflammatory protein 1 alpha (MIP1α), chemokine (C-C motif) ligand 2 (CCL2, also known as MCP1), interferon gamma (IFN-γ), and tumour necrosis factor alpha (TNF-α), which are mainly observed in ICU patients rather than in non-ICU patients (33, 44–49). This signifies a cytokine storm characterised by an abnormal overreaction of the body’s immune system that causes a loss of communication between the infected cells and the host immune defence mechanism. Cytokine storm triggers severe inflammation and infiltration of neutrophils, macrophages, and T cells that may damage several tissues leading to multi-organ failure (50). Carveli et al. (51) demonstrated an association between COVID-19 mediated inflammation and activation of the C5 complement factor with its receptor called complement component C51 receptor (C5AR1). C5AR1 or C5a is a G-protein coupled receptor that modulates inflammatory response by activating neutrophils and monocytes to the site of damage.

#### Invasion of SARS-CoV-2 in Secondary Organs

**Figure 2:**
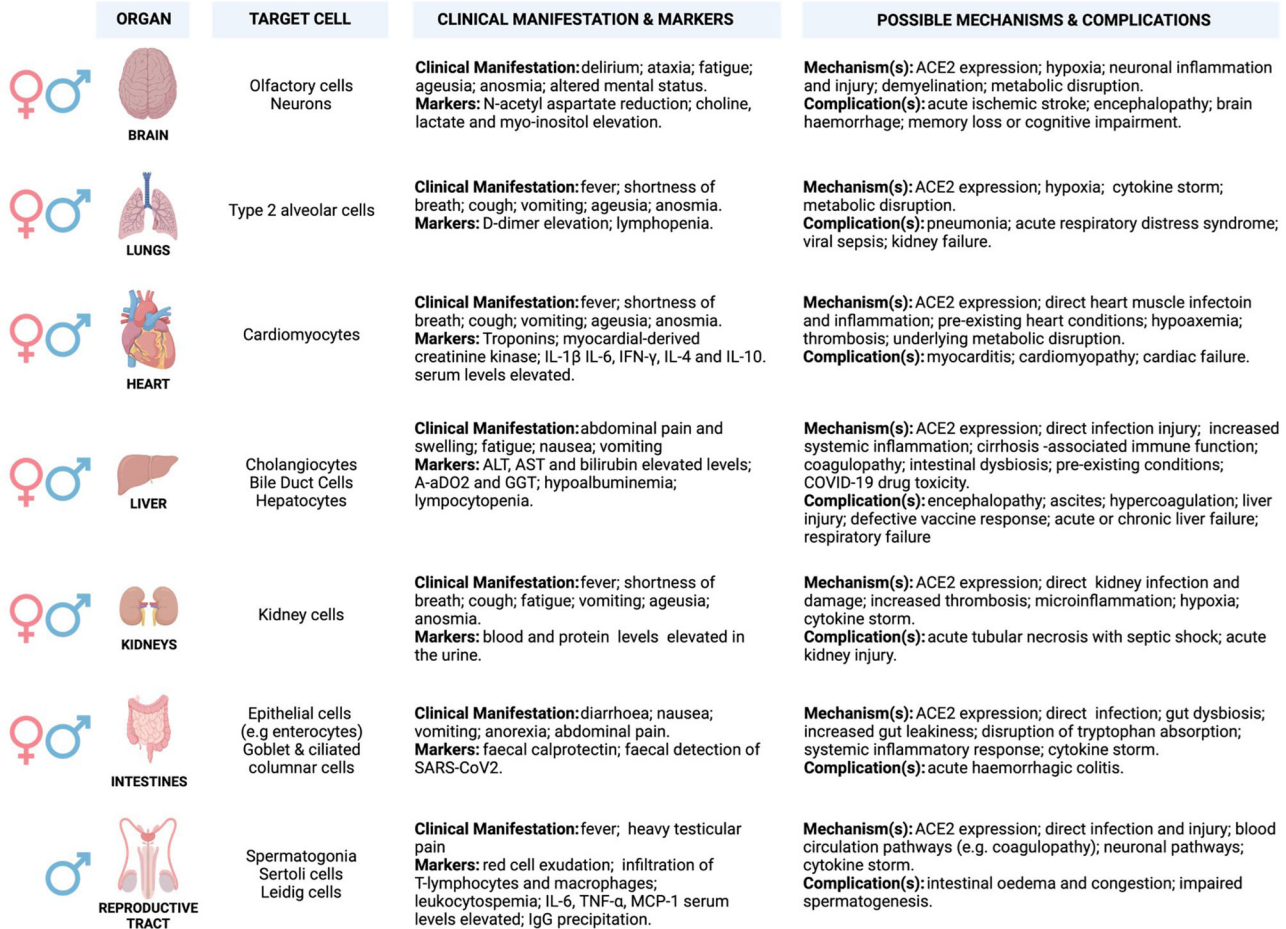
Potential underlying mechanisms of SARS-CoV2 invasion and multi-organ induced damage. Inflammation mediated by SARS-CoV-2 infection and its primary receptor ACE2 drive multi-organ failure in severe COVID-19 cases. ACE2 is widely expressed in multiple organs, and its suppression may aggravate COVID-19 severity and negatively impacts multiple organs via regulation of RAS. Moreover, this leads to severe cases of COVID-19 that are often associated with ARDS and increased mortality rate, partially mediated by the overproduction of pro-inflammatory cytokines (cytokine storm). Cytokine storm results from increased levels of inflammatory mediators, endothelial dysfunction, coagulation abnormalities, and infiltration of inflammatory cells into the organs. This may be characterised by elevated levels of interleukin-6 (IL-6), nuclear factor kappa B (NFκB), and tumour necrosis factor-alpha (TNFα) released from SARS-CoV-2-infected macrophages and monocytes. The involvement of different organs in severe patients is characterised by multi-organ failure and a broad spectrum of haematological abnormalities and neurological disorders that lengthen the hospitalisation duration and increase mortality. The most important mechanisms are related to the direct and indirect pathogenic features of SARS-CoV2 infection. (Created with BioRender.com). ACE2, Angiotensin I-converting enzyme-2; AoDO2, First alveolar-arterial oxygen gradient; ALT, Alanine aminotransferase; AST, Aspartate aminotransferase; IFN-γ, Interferon-gamma; IL-1β, Interleukin- 1β; IL-4/6/10, Interleukin- 4/6/10; TNF-α, Tumour necrosis factor-alpha; MCP-1, Monocyte chemoattractant protein-1.

ACE2 is widely expressed in a heterogeneous population of systemic cells (**Figure 2**), making it possible for SARS-CoV-2 to damage several systemic tissues leading to various clinical phenotypes that result in multi-organ dysfunction (**Figure 2**) (52–61). A high level of ACE2 in nasal epithelial cells correlates with increased viral load, especially in the early stages of SARS-CoV-2 infection (62). This may explain the accuracy of nasal and nasopharynx aspirates for SARS-CoV-2 diagnosis (62). The highest viral load was reported in the olfactory epithelium, suggesting damage in the supporting cells (61, 63–66). Although ACE2 level is low in the capillary endothelial cells of the cerebral circulation, circumstantial evidence suggests that SARS-CoV-2 may access these cells by crossing the blood-brain barrier, as demonstrated by *in vitro* studies. This may involve unknown indirect mechanisms that may be responsible for clinical manifestation (examples are anosmia, ageusia, and altered mental status) and neurological complications that have been observed in critical cases of COVID-19 infections (**Figure 2**) (67–73).

ACE2 and TMPRSS2 are also expressed in cardiomyocytes, cholangiocytes, hepatocytes, and enterocytes, suggesting potential targets for SARS-CoV-2 infection (60, 74–76). ACE2 synergises with the RAAS to regulate angiotensin to balance the normal function of the cardiovascular system (77, 78). Upon SARS-CoV-2 infection, ACE2 is suppressed and fails to counteract the vasoconstrictive and pro-inflammatory function of the RAAS to balance the system. This may lead to increased vascular permeability, tissue oedema/damage, and systemic microcirculatory dysfunction associated with cardiovascular-related disease (79). Approximately 50% of COVID-19 hospitalised patients exhibit abnormal levels of alanine transaminase (ALT) and aspartic transaminase (AST), slightly elevated level of bilirubin, higher alveolar-arterial oxygen gradient (A-aDO2)/gamma-glutamyl transferase (GGT), and hypoalbuminemia that suggests hepatic damage (80–82). Elevated levels of ALT (7590 U/L) and AST (1445 U/L) were almost doubled in severe/critical cases as relative to mild/moderate cases, and correlate with nausea, vomiting, and anorexia (83–85). In addition, a sub-group of COVID-19 patients present with darkened faces and pigmentation (86, 87). This may suggest abnormal liver function probably from failing to metabolise oestrogen, increased iron level, and melanin secretion as well as adrenocortical hypofunction associated with hepatic injury (88–91).

Zhao et al. (92), demonstrated that SARS-CoV-2 infection triggers direct cholangiocytes damage by perturbing the barrier and bile acid transporting functions of cholangiocytes *via* abnormal regulation of *solute carrier family 10-member 2* (*SLC10A2*) gene and *cystic fibrosis transmembrane conductance regulator* (*CFTR*) gene, resulting in bile acid accumulation and consequent hepatic injury aggravation. Mechanisms associated with COVID-19-related hypoxia, antiviral drugs/incorrect drug dosage, and use of herbs or traditional medicines to counteract COVID-19 effects may also participate in liver injury (93–99). Numerous studies have reported successful isolation of SARS-COV-2 from faecal/stool samples of COVID-19 patients with and without inflammatory bowel disease (IBD) (100–103). Interestingly, in some COVID-19 cases, the faecal viral load was even higher (10^7^ copies/g) than in pharyngeal swabs (101, 104). This observation disputes the pharyngeal infection as the source of faecal viral RNA and supports the theory of enteric infection of SARS-CoV-2 (101, 104). An elevated level of faecal calprotectin, largely expressed by neutrophils and a reliable faecal biomarker of intestinal inflammation, has been reported in COVID-19 patients with diarrhoea as compared to patients without diarrhoea (105).

It has been demonstrated that nephrons, undifferentiated spermatogonia, testicular Sertoli, and Leydig cells express a considerable abundance of ACE2 receptor expression, making the kidney and testes further potential SARS-CoV-2 reservoirs (106, 107). Renal damage in cases with no underlying renal conditions suggested SARS-CoV-2 as the underlying cause, and this was marked by abnormal blood work and increased levels of proteins in the urine. Lengthy hospitalisation stays, acute kidney injury (AKI), and increased mortality were the most common consequences of severe or critical cases of COVID-19 (107–113). COVID-19 causes severe physiologic and neurological stress, which may release increased stress hormone and alter testosterone levels. Testes play an important role in regulating the hypothalamic-pituitary-testicular (HPT) axis, which governs the male reproductive hormonal cascade (114). HPT axis endocrinologically links testes to the brain by gonadotropins (luteinising hormone-LH and follicle-stimulating hormone-FSH) and testosterone. LH and FSH that normally activate Leydig and Sertoli cells, respectively, are altered in COVID-19 patients, and this is hypothesised to be due to imbalances in testosterone production (115–118). Levels of LH seem to increase in male patients with severe COVID-19 leading to abnormal FSH/LH ratios (115, 116).

A recent case report of semen analysis for *in vitro* fertilization procedure revealed that mild COVID-19 infection in men could result in long-term alterations in sperm morphology and sperm DNA integrity that may ultimately lead to male infertility (119). It was previously thought that the sperm parameters would take 70 – 90 days to return to their basal state after recovering from the infection. However, this published case has shown that this can take a much longer time of >4 months (119). Although these findings are based largely on case studies and lack further validation, it is plausible to hypothesize that increased risk of infertility as a COVID-19 long-term complication, especially in young men, will be observed after the pandemic. Therefore, more studies are needed to determine the negative impact of COVID-19 in a large cohort of infected males with varying severity of disease during infection and after recovery.

## The Role Of Epigenetics In Infection Susceptibility: X-Chromosome Inactivation And Covid-19

**Figure 3:**
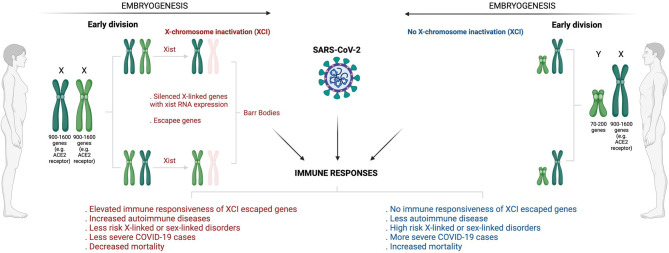
Overview of sex-based differences in the immune response to COVID-19. The diagram shows how X-chromosome inactivation escapee genes may underlie sex bias differences in COVID-infection, severity, and mortality. Sex-bias differences in COVID-19 may be linked to ACE2, the primary receptor that enables SARS-CoV-2 infection. Having double X-chromosomes protects women against increased susceptibility to COVID-19 infection and associated severe complications as compared to men who have just a single X-chromosome. ACE2 is an X-chromosome-linked gene that escapes X-inactivation, a phenomenon that suppresses gene transcription from one of the two X chromosomes in female mammalian cells to balance expression dosage between XX females and XY males. This means that women have twice more genetic instructions to transcribe ACE2 and many more X-chromosome-linked immunoregulatory genes that protect women from increased COVID-19 susceptibility and associated severe complications. (Created with BioRender.com). ACE2, Angiotensin I-converting enzyme-2; COVID -19, Coronavirus disease 2019; SARS-CoV-2, Severe acute respiratory syndrome coronavirus 2; XCI, X-chromosome inactivation; XIST, X-inactive specific transcript.

In terms of Betacoronavirus (SARS-CoV-2, severe acute respiratory syndrome coronavirus/SARS-CoV and Middle East respiratory coronavirus/MERS), men usually experience severe infections complicated with poorer clinical outcomes than women (120–124). It was observed that SARS-CoV-1 infected males had a significantly (21.9%, p < 0.0001) higher case fatality rate than females (13.2%) with a relative risk of 1.66 (95% confidence interval (CI): 1.35, 2.05) before age adjustment and 1.62 (95% CI: 1.21, 2.16) after adjustment (120). Peckham et al. (123), demonstrated through a meta-analysis of 3,111,714 reported global COVID-19 cases that males have almost three times the odds of requiring ICU admission (OR = 2.84; 95% CI = 2.06, 3.92) and higher odds of fatality (OR = 1.39; 95% CI = 1.31, 1.47) compared to females. X-chromosome inactivation (XCI) may explain some of the disparities in infection susceptibility (**Figure 3**). As an epigenetic hallmark of normal human development, XCI is regulated by a progressive and stepwise epigenetic phenomenon that ensures an equal dosage compensation of the X-chromosome encoded genes expression level between females and males (125, 126). XCI is regulated by the X-inactivation centre (XIC) and established by long non-coding X inactive specific transcript (Xist) RNA through several heterochromatin changes as largely demonstrated by seminal work conducted by the Brockdorff lab (127, 128). The suppression of X-linked genes through recruitment of the PRCs is a common XCI feature (129). Acquisition of histone deacetylase 3 (HDAC3) and H2A by adding a single ubiquitin group to lysine-119 (H2AK119) are the earliest repressive epigenetic marks required for efficient XCI. H3K27me3, a transcriptional silence mark that is catalysed by PRC2-EZH2 for inactive heterochromatin, is enriched and later spread at the promoters of silenced X-linked genes for long-term stable XCI maintenance (129, 130).

Notably, for counteracting invading pathogens, the X-chromosome is enriched with many immune-related genes and regulatory elements that activate host immune defence mechanisms (131). While this may increase women’s susceptibility to autoimmune disease, it may also provide them with immunological and survival advantages against pathogen insults (132). Females have two copies of X-chromosome (XX), and one becomes randomly and permanently silenced during embryogenesis through XCI (125, 126). An inactivated chromosome is called a Barr body or sex chromatin (**Figure 3**). Some genes located in the silenced X-chromosome may escape XCI and remain expressed to perform their normal activities (133). Fortunately for women, these XCI skewing genes/escapees may lead to an elevated level and high immune responsiveness of such genes (134). Subsequently, this results in double and exclusive protection for women against defective X-linked genes and infections relative to men (**Figure 3**). As a result of having a single copy of X-chromosome (XY), males are at high risk of X-linked or sex-linked disorders (134), and this may explain why males tend to suffer more severe cases of COVID-19/other infections and fatal complications than females. Sex different effects in COVID-19 may be attributable to various external risk factors that are more prevalent in men *versus* women (135–140). Comorbidities such as cancer, heart failure, hypertension, diabetes, obesity and chronic obstructive pulmonary disease coupled with behavioural factors including smoking and alcohol consumption are generally increased in males than females, and these have been shown to correlate with poor clinical outcomes, increased risk of ICU admission and fatalities in COVID-19 infected patients (135–138, 140, 141). Men have been shown to have an increased level of circulating plasma ACE2 receptor, the primary receptor that enables SARS-CoV-2 attachment and infection (138, 139, 142). Using a high-throughput multiplex immunoassay based on a proprietary proximity extension assay (PEA) technology, Sama et al. (141) measured the ACE2 concentration in index cohort of 1485 males and 537 females with COVID-19 and heart failure, and found that the mean plasma concentration of ACE2 was higher by 5.38 in males compared with females (5.09, *P* < 0.001). This was also supported by a validation cohort that exhibited increased 5.46 ACE2 plasma concentration in males compared with 5.16 in females patients (*P* < 0.001) (141). A separate single center population-based study of 5457 Icelanders demonstrated altered serum levels of ACE2 in males, smokers and diabetes or obese patients, and this was associated with productive SARS-CoV-2 infection and severe clinical outcome (142). The expression levels of ACE2 receptor was found to be enhanced in the lungs in response to active smoking, diabetes and hypertension, explaining an increased susceptibility and severity to COVID-19 infection (138, 139, 142).

Gene expression regulation of ACE2 and other X-chromosome linked genes, including Toll-like receptors (TLRs), CD40 ligand (CD40L), and Forkhead box P3 (FOXP3)/Scurfin, expressed upon SARS-CoV-2 infection, may play a critical role in COVID-19 pathogenesis and severity. Following viral entry, SARS-CoV-2 triggers the activation of the RNA-based pathogen sensors such as TLR3, TLR4, TLR7, and retinoic acid-inducible gene-I-like receptors (RIG-I), which complex with a melanoma-differentiation associated 5 (MDA-5) to establish a frontline defence mechanism (143). This complex is epigenetically subverted to induce abnormally elevated levels of interferons (IFNs) and pro-inflammatory cytokines, such as tumour necrosis factor alpha (TNF-α) and interleukins (ILs), associated with critically ill and ICU admission of COVID-19 patients (131, 144).

Dai et al. (145), through integrated bioinformatics analysis revealed an upregulation of structural maintenance of chromosomes flexible hinge domain containing 1 (SMCHD1) in COVID-19 patients, suggesting that it may be involved in the epigenetic control of ACE2 receptor, and thus COVID-19 pathogenesis. It is not surprising that SMCHD1 is linked to ACE2 receptor regulation, as it is an essential protein in XCI. Mouse studies have demonstrated that homozygous nonsense mutations in the *Smchd1* gene cause XCI defect that leads to female-specific embryonic lethality (146, 147). Gendrel et al. (125), demonstrated that a late step *Smchd1* gene recruitment to XCI in female XX embryonic stem cells establishes DNA methylation of CpG islands, preferably *via Dnmt3b* gene and histone mark H3K27me3 for long-term maintenance of gene silencing. An SMCHD1-dependent pathway may explain the data of Mudersbach et al. (148), demonstrating that TNF-α suppresses ACE2 mRNA and its protein expression in endothelial cells *via* hypermethylation by DNMTs, including *DNMT3b*. It has been suggested that suppression of TNF-α mediated ACE2 mRNA *via* epigenetic inhibitors may reduce SARS-CoV-2 viral replication, leading to anti-inflammatory effects associated with quicker healing and resolution of COVID-19-related complications (148). SARS-CoV-2 genome encodes mRNA Cap 2´-O-Methyltransferase (2-O-MTase), another epigenetic phenomenon that deposits a methyl group at the 2´-O position of the first nucleotide adjacent to the cap structure at the 5’ end of the RNA (149–152). RNA-based viruses often use this mechanism to their advantage to escape immune surveillance. It might be tempting to speculate that drugs targeting these epigenetic marks and preventing immune evasion may also be important in fighting COVID-19 infection.

## Possible Epigenetic Dynamics In Covid-19 Infection

Li et al. (153) have demonstrated in a murine mouse model with the human ACE2 (hACE2) transgene that SARS-CoV-2 induces epigenetic-mediated metabolic reprogramming and alterations in both local and systemic sites of infection. These alterations are associated with systemic lethality that mirrors human COVID-19 clinical phenotypes, suggesting an epigenetic role in COVID-19 pathogenesis. Below, we discuss epigenetic marks and alterations that we hypothesize may play a role in ACE2 receptor regulation and COVID-19 pathogenesis/treatment.

### Writing of DNA Methylation and Role of DNMTs

DNMT1, DNMT3A, DNMT3B, and DNMT3L are family of DNMTs that write or deposit methylation on DNA leading to hypermethylation, read by MBDs to mainly suppress gene transcription (**Figure 1**) (4, 154). DNMT1 binds to and methylates hemi-methylated CpG sites to ensure stable maintenance of DNA methylation (4). DNMT 3A and 3B are *de novo* methyltransferases that mainly lead to transcriptional repression through the establishment of non-CpG methylation, an emerging epigenetic mark that defines brain tissue-specific patterns of gene transcription (155–159). DNMT3L is catalytically inactive and serves as a cofactor for DNMT 3A and 3B (160, 161).

Although DNA methylation patterns are erased and deposited through successive normal developmental stages and cell differentiation, they also occur in the form of epigenetic memory in stem cells, and in communicable and non-communicable diseases, reviewed in (162). Most importantly, various epigenetic phenomena triggered in response to raging viral replication are usually hijacked by the same targeted virus to alter the protective immunoregulatory mechanisms for survival and propagation, reviewed in (163). For instance, during infection with hepatitis B virus (HBV), DNMTs are upregulated in response to productive viral replication mediated by the host-viral interaction as part of host immune defence mechanisms, also reviewed in (164). In the long run, the same DNA methylation machineries may start hypermethylating CpG island promoters that overlap with host-viral integration sites leading to alteration in the transcription of genes, including immunoregulators and tumour suppressors that are critical to carcinogenesis (164). COVID-19 related airborne respiratory infections such as the Middle East respiratory syndrome-CoV (MERS-CoV) and avian influenza (H5N1) have also been shown to exploit DNA methylators and histone modifiers to suppress immunoregulators such as type 1 IFN-γ-responsive genes. These genes include *class II, major histocompatibility complex, transactivator* (*CTIIA*), *antigen peptide transporter 2* (*TAP2*), and *protein disulfide-isomerase A3* (*PDIA3*) (165). Abnormal regulation of these genes impedes the host immune system to fight infections effectively (166). This suggests that various epigenetic reprogramming phenomena may also occur during COVID-19 infection (167).

Mice transfected with hACE2 and subsequently infected with SARS-CoV-2 have been used to gain insights into epigenetic changes that drive cardiac injury in COVID-19 patients (153, 168). Li and colleagues identified 172 differentially methylated CpG sites in the hearts of SARS-CoV-2-infected mice compared with controls (153). Two genes, *paternally expressed gene 10* (*Peg10)* and *endothelin-converting enzyme 1* (*ECE1*), show high levels of differential methylation in SARS-CoV-2 mice bearing hACE2 compared with controls. For the *Peg10* gene, a hypomethylation pattern consistent with higher expression of the *Peg10* gene in hearts was seen. The loss of function of the *Peg10* gene is known to result in early embryonic death (169). *Peg10* gene also regulates cellular proliferation and viral replication through binding to the viral transcription regulators (170). SARS-CoV-2 infection was associated with increased methylation of the *ECE1* gene, the product that regulates proteolysis of endothelin precursors to form biologically active peptides (171). Loss of function of the *ECE1* gene is associated with cardiac defects, generalized oedema, and autonomic dysfunction (172). In another study, blood samples from acute SARS-CoV-2 infection *versus* healthy controls blood samples exhibited 28% of hypermethylated regions (173). Hypermethylated regions comprised of more than 5 consecutive differentially methylated CpG sites. It is not surprising that studies with SARS-CoV-1 and MERS also detected differentially methylated CpG sites, and found to be located in the promoter regions encoding genes involved in interferon and antigen presenting cells stimulation (174). This supported a recent study that identified >40 CpG sites encoding genes serving similar purposes, suggesting the role of DNA methylation influencing COVID-19 progression and target for epigenetic therapy (175).

Activation of the immunoregulatory cytoplasmic transcription factor aryl hydrocarbon receptor (AHR) may also result in hypermethylation that contributes to COVID-19 pathogenesis. The AHR has been identified as a host factor for Zika and Dengue viruses, and its inhibition was associated with significantly reduced viral replication and amelioration in the disease pathology (176–178).

It has been shown that the AHR becomes activated upon SARS-CoV-2 infection (178), and that it impacts SARS-CoV2 antiviral immunity and pathogenesis, promoting a pro-inflammatory response and participating in the severity of COVID-19 (178). Furthermore, it has been postulated that AHR activation may be the culprit behind the COVID-19-mediated cytokine storm (145, 178, 179). RNA-Seq analysis of CoV-infected cells unveils an upregulation of the AHR and its target genes, including AHRR and CYP1A1 (177). Kynurenic acid, a product of normal metabolism of L-tryptophan, and a potent endogenous AHR ligand, has also been shown to be elevated in response to COVID-19 (139, 180). This correlated with cytokine storm, age and low levels of T-cell responses, especially in males as compared to female patients, hinting for a sex-specific link to immune response and COVID-19 clinical outcome (139). Curiously, activation of the AHR has been associated with hypermethylation in acute lymphoblastic leukaemia (ALL) *in vitro*. When demethylated by methylation inhibitor zebularine, AHR-related methylation inhibition restored normal cells phenotype and prevented tumorigenesis (181). In another study, AHR activation resulted in epigenetic alteration of Foxp3 and IL-17 expression and consequently attenuated colitis (182). Recently, Jiadi et al. (183), have shown that macaques infected with SARS-CoV-2 modulation of the AHR upregulates the expression of ACE2 by binding to its promoter regions, and this is accompanied by aggressive disease. Consequently, if the AHR becomes hypermethylated, as shown in other pathologies, the level of ACE2 may also be silenced through the same methylation. This may disrupt the inhibitory mechanisms regulated by ACE inhibitors or other RAAS blockers leading to the aggressiveness of underlying cardiovascular diseases (e.g., hypertension) that have been reported in severe/critical cases of COVID-19 infection.

Interestingly, the AHR also regulates the expression of NOD-, LRR- and pyrin domain-containing protein 3 (NLRP3), that may also be epigenetically regulated (184, 185). Castro de Moura et al., revealed a strong correlation between COVID-19 clinical severity and DNA methylation of 44 CpG sites with >50% of these located in 20 promoters of annotated coding genes including *Absent in Melanoma 2* (AIM2) and *major histocompatibility class 1C (HLAC)* (175). *AIM2*, similarly to the NLRP3, is part of the inflammasome complex (186). The inflammasome is involved in caspase-1 cleavage, trigger of gasdermin D-mediated pyroptotic cell death and release of pro-inflammatory cytokines IL-1β and IL-18 in response to pathogens’ insult, reviewed in (187, 188). Altered levels of IL-1β and IL-18 cytokines were observed in COVID-19, as it does in several male infertility-related disorders such as varicocele (49, 111, 189), suggesting that NLRP3 may also be activated upon COVID-19 infection. This notion is supported by the study of Su et al. (190), that demonstrated that upregulation of calcium-sensing receptor (CaSR) activates the NLRP3 pathway in testicular macrophages and impairs testosterone synthesis in a uropathogenic *Escherichia Coli* (UPEC) rat orchitis model.

Chronic infections such as HBV, hepatitis C virus (HCV), and human immunodeficiency virus (HIV) have been demonstrated to infect sperm cells and trigger oxidative stress. Subsequently, this activates histone modifications leading to long-term effects on male fertility parameters such as sperm integrity, count, motility, and morphology. During normal differentiation, sperm cells’ genome undergoes successive rounds of epigenetics marks to ensure proper spermatogenesis and spermiogenesis (191). More than 85% of human mature sperm cells’ DNA is bound to protamines. Protamines are sperm-specific basic nuclear proteins that take over the histones’ position and function to package the sperm DNA for compaction necessary for sperm motility (192). In the late stages of spermatogenesis, sperm cells’ genome becomes dramatically reorganised and globally hyperacetylated to remove and replace histones with protamines. This phenomenon essentially erases the epigenetic modifiers laid out through histone modifications. It preserves the paternal genome by protecting it from extracellular stressors and harmful effects of the oocyte during fertilisation (192). An altered protamine ratio or histone content or distribution in sperm is a sign of aberrant chromatin packaging, associated with increased susceptibility to DNA damage or abnormal epigenetic marking that may lead to male infertility. Ma et al. (108), have detected SARS-CoV-2 in the testis’ biopsies of COVID-19 patients. Immunohistochemistry analysis revealed a significant increase in spermatogenic epithelial shedding in the deceased patients with critical cases of COVID-19, which was accompanied by thinning of seminiferous tubules (193). Inflammation of the epididymis and/or testicle was associated with old age (>80yrs) and severe or critical cases of COVID-19 (P = .037) (107). More than 20% of recovered patients who previously had children through natural birth exhibited autoimmune orchitis. This was indicated by observed oligospermia, leukocytospermia, elevated sperm phagocytic CD3+/CD68+ immune responses in testes/epididymis and apoptotic cells relative to age-matched control males (193). In some cases, tocilizumab was administered in response to a progressive worsening of oxygenation, and blood biochemistry tests revealed an elevation of lactate dehydrogenase to 1213 U/l, D-dimer to 1150 ng/ml, and CRP to 23.80 mg/dl. Impaired spermatogenesis and increased apoptotic cells may be attributable to COVID-19-induced histone modifications associated with elevated CRP and fever that perturbed the optimum testicular temperature (2 – 4 ⁰C below the average body temperature) (194). Moreover, extensive germ cell destruction, as demonstrated by the TUNEL assay, may have also been a contributing factor.

### Erasing of DNA Methylation: Role of TETs

TETs are regarded as erasers of DNA methylation, reviewed in (195). They actively or passively demethylate DNA methylation by removing the 5-methylcytosine mark. TETs oxidise 5-methylcytosine to generate 5-hydroxymethylcytosine (5-hmc), 5-formylcytosine (5-fc), and 5-carboxycytosine (196–198). 5-hmc is a stable epigenetic mark that is highly abundant in the brain, liver, and stem cells, and it is crucial for neurogenesis and hepatocellular carcinoma (HCC) (199, 200). TETs are prominent regulators of immune cells. For example, Tet-2 mediates T-cell differentiation and synergises with Tet-3 to modulate the expression of Foxp3, a transcription factor responsible for T-cells development (201). Tet-deficient mice CD4-T cells exhibited impaired Th1/2/17 differentiation and cytokine production in lymphocytic choriomeningitis virus infection, supporting a critical role of Tet-2 in infections (202). In other studies, loss of Tet2/3 resulted in an antigen-driven expansion of various immune cells and rapidly developed aggressive disease phenotype (203, 204). Moreover, combined deletion of Tet2/3 in mice exhibited impaired Treg cell differentiation. This was accompanied by DNA hypermethylation of various Treg-specific demethylated regions (TSDRs) within the Foxp3 locus that resulted in aberrant Foxp3 expression (205, 206). TNFs and ILs, important cytokine storm elevated markers observed in severe or critical cases of COVID-19, are known to induce DNA demethylation *via* TETs (207–210). IL-1β and TNF-α modulate the global hydroxymethylation by activating TETs and iso-citrate dehydrogenases in the genomic DNA and specific locus in matrix metalloproteinase (MMP) promoter region in human OA chondrocytes (211).

In severe COVID-19 pneumonia cases, abnormal upregulation of T-cell proliferation, activation, and cytotoxicity was noted at the late phase of infection, suggesting an underlying perturbation resulting in the loss of an inhibitory role Tregs (212). Mohebbi et al. (210), have shown that CD4+ FoxP3+ CD25+ T cells expression level is significantly suppressed in hospitalised COVID-19 patients and led to an elevated level of IL-6. Given this evidence, it is intriguing to suggest this aberrant hyperactivation of cytotoxic cells in COVID-19 may be attributable to Tet-2/3-mediated epigenetic regulation of Tregs. Cell division occurring as a result of antigen and cytokine stimulation in response to COVID-19 infection may be the underlying mechanism for this epigenetic reprogramming. This may result in aberrant gene transcription, fatal inflammatory response, disease aggressiveness, and multi-organ disease phenotypes observed in severe and critical cases of COVID-19.

Abnormal production of the IFN and IFN-γ correlate with slowly resolved COVID-19, and enhanced viral replication was also observed, as previously reported in other studies (213–215). This may also correlate with genetic variation of heat shock protein 70 (HSP70) or A1L (HSPA1L), which has been demonstrated to result in significantly higher plasma concentrations of TNF-α and IL-6 and poor clinical outcomes after severe tissue injury from pathogens (216). Elevated levels of TNF-α and IL-6 are associated with severe cases of COVID-19 and systemic inflammation, as well as *HSPA1L* gene upregulation *via* hypomethylation of its promoter regions in response to increased SARS-CoV-2 viral replication (217). *HSPA1L* hypomethylation is catalysed by the dramatically reduced DNA methyltransferases (DNMT 1 - 3), possibly *via* TETs and postulated to enable viral cell entry and protein synthesis (217, 218).

### Writing Histone Modification: Role of HATs and HMTs

Histone lysine acetylation is catalysed by conserved histone acetyltransferases (HATs) and plays a crucial role in viral infections (219, 220). It facilitates the transfer of an acetyl functional group from acetyl coenzyme A to the ε-amino group of the lysine residue at one end of the histone molecule on the chromatin. HATs alter the charge of various lysine residues within either H3 (histone acetylation at lysine 9, 14, 18, and 23, denoted as H3K9/14/18/23ac) or H4 (H4K5/8/12/16ac), reviewed in (221–223). A positive charge from lysine becomes neutralised by a negative charge from a transferred molecule, reducing the binding affinity between histones and DNA. This alters the chromatin architecture by opening the chromatin and making it accessible to the transcription factors for active gene expression (221–223). MYST writes histone acetylation, adenoviral E1A-associated protein of 300 kDa/CREB-binding protein (p300/CBP) and general control non-derepressible 5 (GCN5)-related N-acetyltransferases (GNATs) and read by bromodomains (BRD) and extra-terminal (BET) family of proteins (221, 222).

Histone H3 and H4 form a significant component of the host immune defence mechanism against pathogen insults and other hostile environments. *In vitro* studies with retroviral infected mouse embryonic fibroblasts have shown that histones are loaded rapidly on unintegrated retroviral DNA soon after infection (219, 220). Unintegrated retroviral DNA is typically weakly expressed, but in response to interaction with loaded histones, their expression may become dramatically increased by chromatin modifiers and promote persistent infection (219, 220). Several studies have shown that histones can be released into blood circulation during an infection as damage-associated molecular patterns (DAMPs) from apoptotic and damaged cells, eliciting an inflammatory stimulus (224–226). DAMPS interact with TLRs, and trigger TLR/myeloid differentiation factor 88 (MyD88)/NLRP3 pathways leading to activation of macrophages (227). This, in turn, can cause an accumulation of neutrophil infiltration and subsequent production of neutrophil extracellular traps (NETs) and reactive oxygen species (ROS) (227). Activation of TLR/MyD88/NLRP3 pathways has been upregulated in obese patients that are at high risk of severe COVID-19 infection (228). This suggests that activating these pathways by the DAMPs and histones loaded on viral proteins may be the mechanism underlying an excessive tissue inflammation and injury that correlates with multiple organ failure and increased mortality in COVID-19 infection.

Histones can also bind to complement component 5a (C5a) and CRP, which are proteins expressed by the liver in response to systemic inflammation (229–231). CRP is a regulatory factor for angiogenesis and thrombosis associated with cardiovascular disease (CVD), which is a risk factor for COVID-19 severe cases (232). An elevated level of C5a and CRP in COVID-19 infection is an indication of excessive inflammatory response in endothelial cells and tissue damage that correlates with aggravated disease or poor prognosis. Neutrophils play an important role in the early or later stages of severe cases of influenza A virus (IAV), and COVID-19 infection cases, where circulating cell-free histones are enriched and highly pro-inflammatory (233). Hsieh et al. (233) have shown that binding of histones H4 to CRP in neutrophils models infected with IAV blocks the H4-mediated neutrophil activation and potentiates neutrophil inflammatory response during infection (233). This data suggests that H4 may be part of the host protective mechanism during excessive pro-inflammatory response. However, in response to interaction with circulating virus through molecules such as C5a and CRP, this mechanism may be hijacked by the virus for its replication advantage leading to tissue damage and fatal sequelae observed in COVID-19.

A case study of four unrelated young men who were critically ill with COVID-19 infection, and subjected to mechanical ventilation in the ICU, revealed nonsense and missense X-chromosomal TLR7 variants using whole-exome sequencing (213). This TLR7 variant mutation resulted in a unique loss of function from aberrant alteration of TLR7 mRNA expression and its downstream target genes. *Interferons regulatory factor 7* (*IRF7*), *interferon beta 1 (IFNB1*), and *interferon stimulated gene 15* (*ISG15*) are examples of genes associated with this TLR7 variant mutation. IRF7 becomes acetylated by HATs p300/CBP-associated factor (PCAF) and GCN5, and this usually impairs its binding activities leading to reduced IRF7 activity. PCAF acetylase complex and GCN5 are required for viral integration, and they have also been shown to be activated in influenza A virus to negatively regulates the viral polymerase activity (234, 235). PCAF is also known as lysine acetyltransferase 2B (KAT2B), a master regulator of TGF-β signalling pathway that triggers CVD development when altered. The SARS-CoV-2 virus induces an aberrant and excessive TGF-β-mediated chronic immune reaction creating a switching from IgM to IgA1 and IgA2 immunoglobulins (38, 236). This, in turn, causes an increased pro-inflammatory response and severe disease activity that correlates with prolonged ICU COVID-19 cases and fatalities (38, 236). It is important to investigate the possible roles of PCAF and GCN5 activities in regulating TGF-β and TLR7 signalling pathways in severe COVID-19 for novel treatments to ameliorate the severity and prevent COVID-19 fatalities.

Unlike histone acetylation, histone methylation does not modify any histone protein charge but deposits one or a set of methyl groups from S-adenosyl methionine (SAM) on the side chains of either H3 or H4 lysines or arginine (237). Histone methylation is catalysed by histone methyltransferases (HMTs) with various methylation sites (238). One of these HMTs is SET1B with H3K4me3 occupancy on open chromatin, and this recruits transcription factors for epigenetic transcriptional activation (239). This epigenetic tag has been shown to induce hypoxia, one of the emerging key drivers of COVID-19 pathogenesis and related fatalities. COVID-19 related-hypoxia manifests insufficient levels of oxygen supply in various tissues. SET1B activation is oxygen-dependent and facilitates hypoxia responses *via* site-specific histone methylation (240). In response to hypoxia, SET1B is recruited to the hypoxia-inducible transcription factor (HIF) promoter *via* HIF1α and facilitates the expression of genes involved in angiogenesis (240), one of the clinical features of COVID-19 severity. HIF-related genes will be described further in a later section of histone demethylation.

### Erasing Histone Modification: Role of HDACs and LSDs/KDMs

Histone acetylation and methylation are erased by HATs and lysine demethylases (KDMs)/lysine-specific demethylases (LSDs), respectively. The former result in a more condensed, closed, and transcriptionally silenced chromatin structure that is not accessible to transcription machineries (6–10). The latter blocks the recruitment or occupancy of transcriptional factors on the chromatin sites (9). The process is called histone lysine deacetylation or demethylation, and it associates with the repression of gene transcription. HDACs are grouped into four classes, including class I (HDACs 1 - 3 & 8), class II (HDACs 4 -7, 9 & 10), class III (Sirtuin 1 - 7) class IV (HDAC 11), reviewed in (241, 242). KDMs/LSDs include KDMs/LSDs 1 – 6 with several families that act on different substrates for various cellular processes.

Histone repression marks are common phenotypical features in viral infections and other diseases, such as cancer (243). Virus-induced cancers from HBV, HPV, and EBV hijack histone acetylation marks for viral survival and propagation, and various HDACs inhibitors have been shown to circumvent these effects and alleviate the disease (244–247). Sirtuin 1 (SIRT1) is a key epigenetic regulator of CVD, metabolic and age-related disease through interaction with nuclear transcription factor-κB (NF-κB), a master regulator of inflammation activated by a signal transducer and activation of transcription 3 (STAT3) (248, 249). STAT3 becomes hyperactivated and impairs immune defence machineries that promote exacerbated inflammation and lymphocytopenia, leading to lung fibrosis and thrombosis, as demonstrated in severe COVID-19 cases (249). SIRT1 was also shown to interact with and modulate p53 activities to regulate viral replication in MERS-CoV and SARS-CoV infections (250, 251). Takahashi et al. (252), have recently shown that panobinostat, an inhibitor that counteracts HDACs effects, silenced the transcription of ACE2 receptor and ABO gene (gene encoding three blood group alleles) in cultured epithelial cell lines. This suggested a potential preventative drug against COVID-19 infection (252, 253). ACE2 is the primary host receptor for viral entry, whereas the ABO blood group system has been suspected to increase susceptibility for severe COVID-19 cases (252, 253). Related to this, Zhao et al. (254), have shown that blood group A individuals may be susceptible to COVID-19 infection, owing to the enrichment of group A antigen in respiratory cells (254).

**Figure 4:**
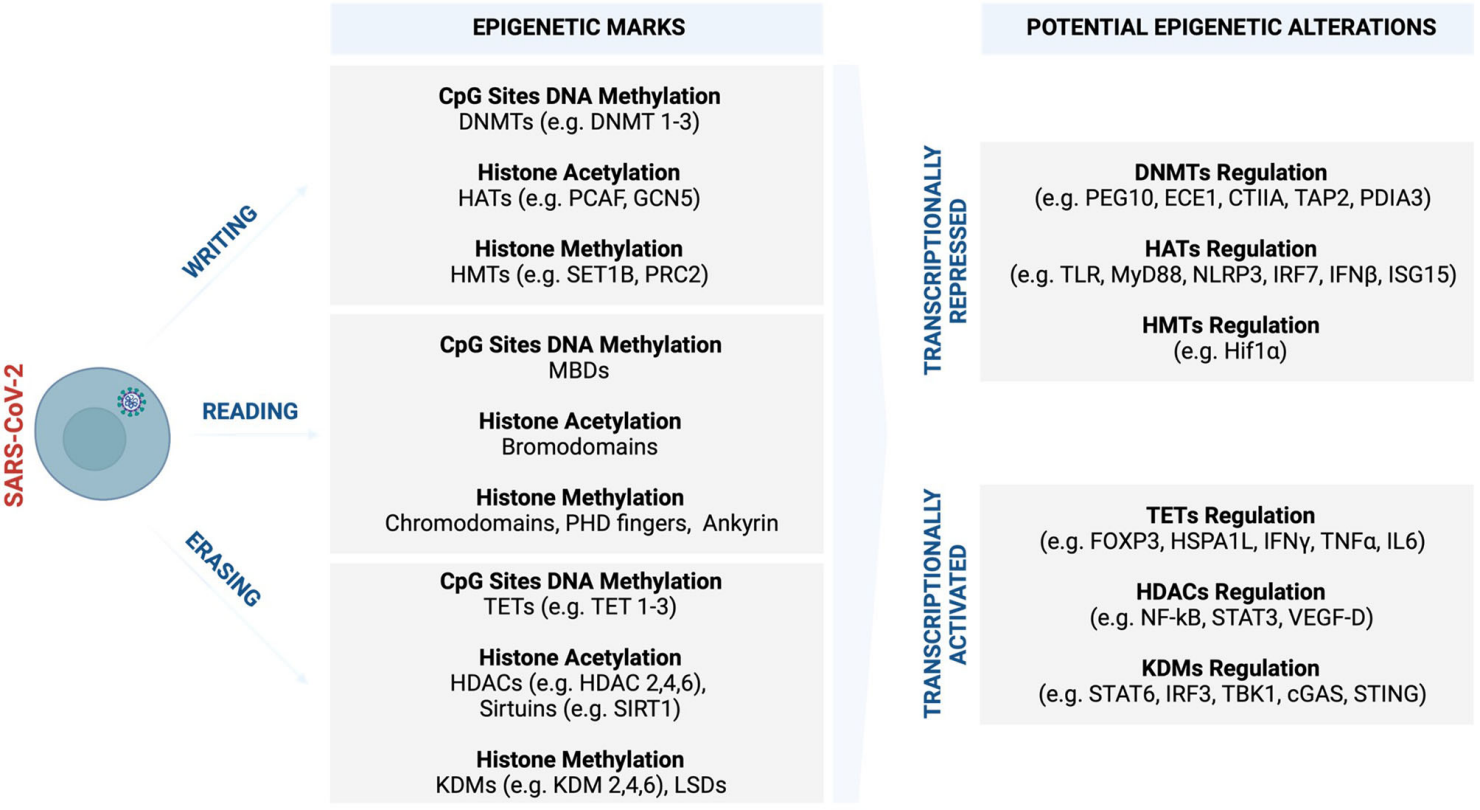
Potential COVID-19 related epigenetic alterations and clinical implications. Like many other viruses, SARS-CoV-2 may trigger various epigenetic alterations such as global DNA methylation and histone modifications, which synergistically cooperate in influencing and driving the course of COVID-19 and sex-bias differences. In response to increased viral replication, the infected host cells may set off an epigenetic signature to antagonise the virus as part of innate immune defence machinery. Part of this epigenetic landscape may be subverted to benefit the virus and its propagation, leading to enhanced and systemic COVID-19 infection that promotes severe complications. (Created with BioRender.com). cGAS, Cyclic GMP–AMP synthase; CIITA, The major histocompatibility class (MHC) II transactivator; DNMTs, DNA methyltransferases; ECE1, endothelin Converting Enzyme 1; FOXP3, Forkhead box protein P3; GCN5, General control non-repressed 5 protein; HATs, Histone acetyltransferase; HDAC, Histone deacetylase; HIF-1α, Hypoxia-inducible factor-1α; HSPA1L, Heat shock protein family A (Hsp70) member 1-like; IFN-γ, Interferon gamma; IFN-β, Interferon-β; IL-6, Interleukin- 6; IRF2, Interferon regulatory factor 2; IRF3, Interferon regulatory factor 3; ISG15, Interferon-stimulated gene 15; JmjC, Jumonji C; KDM, Histone lysine demethylases; LSD, Lysine-specific demethylases; MBDs, Methyl-CpG binding domains; MyD88, Myeloid differentiation factor 88; MLL, Mixed-lineage leukaemia; NF-κB, Nuclear factor kappa B; NLRP3, NOD-, LRR- and pyrin domain-containing protein 3; PDIA3, Protein disulfide isomerase family A member 3; PEG10, Paternally expressed gene 10; PHD, Plant homeodomain; PRC, Polycomb repressive complex; p300/CBP, p300 and cyclic AMP response element-binding protein; STAT-3, Signal transducer and activator of transcription-3; STAT-6, Signal transducer and activator of transcription-6; SET1, Suppressor of variegation 3–9, enhancer of zeste, trithorax 1; SIRT, Sirtuins; STING, Stimulator of interferon genes; TAP2, Transporter 2, ATP Binding Cassette Subfamily B Member; TBK1, Tank binding kinase 1; TET, Ten-eleven translocation; TLR, Toll-like receptors; TNF-α, Tumour necrosis factor-alpha; UTX1, Ubiquitously transcribed tetratricopeptide repeat, X chromosome 1; VEGF-D, Vascular endothelial growth factor D.

Upregulation of HDACs by hypoxia was shown to be activated in response to a silence in hypoxia-responsive tumour suppressor genes (255, 256). These genes include HIF-1α and vascular endothelial growth factor (VEGF), and their epigenetic-mediated alteration correlates with a dramatic increase of intussusceptive angiogenic features (255). A similar clinical phenotype was observed in the lungs of deceased COVID-19 patients, exhibiting distinctive pulmonary vascular pathophysiologic features in a background of perivascular inflammation and injury, as relative to those of influenza (257). VEGF is a prominent mediator of angiogenesis and is usually involved in wound healing (257). VEGF exerts its activities through VEGFR 1–3, which are targeted and negatively regulated by epigenetics alterations (258). VEGFR3 receptor has two ligands, VEGF-C and VEGF-D, which stimulate angiogenesis. Interestingly, serum levels of VEGF-D were found to be significantly elevated in ICU COVID-19 patients as compared to non-ICU patients, a novel biomarker to trace the progression of disease (258). Current research shows that VEGF and its associated receptors undergo histone deacetylation, suggesting them as potential epigenotherapy targets. It has previously been shown that histone deacetylase 4 (HDAC4) remodels neuronal morphology by altering the transcription signature of VEGF-D (259). Activation of HDAC2 suppresses inflammatory cytokines (e.g., IL-17, **Figure 4**) in pulmonary disease, and this with the disease onset and sometimes with prognosis (260–262). On another note, Ahmad et al. demonstrated that endothelial TLR/MyD88 signalling is regulated by histone deacetylase 6 (HDAC6), contributing to alveolar remodelling architecture and pulmonary inflammation (263). Upregulation of TLR/MyD88 signalling pathway in association with elevated TNF-α and IL-6 was reported in overweight and obese individuals as compared to lean individuals (263). In referral to this observation, Cuevas and co-authors have recently published a brief communication postulating and probing for a research study that upregulation of TLR/MyD88 signalling pathway may contribute to excessive and fatal pro-inflammatory cytokine storm especially in SARS-CoV-2 vulnerable obesity individuals (228). MyD88 was shown to establish and promote CD4 T-cells responses to control viral spread to the central nervous system (CNS) in coronavirus-induced encephalomyelitis (228). Any abnormal regulation of MyD88 signalling already existing in obese individuals and other co-morbidities may impact COVID-19 disease progression leading to more fatalities (228).

The widespread methylation of genes in SARS-CoV-2 infection is associated with the downregulation of genes involved in the regulation of the tricarboxylic acid (TCA) and mitochondrion electron transport chain (153). SARS-CoV-2-induced epigenetic alterations interfere with metabolic processes that are core to generating energy for the myocardium (153). The perturbed metabolic processes restrict the energy required for uncontrolled systemic inflammatory response leading to myocardial injury. Transcriptome analysis studies conducted from patients with hypertension and DM associated with severe COVID-19 cases revealed that ACE2 expression was potentially regulated synergistically by various histone marks such as histone acetyltransferase 1 (*HAT1*), *HDAC2*, and lysine demethylase 5B (*KDM5B)* (264). *KDM5B* is a histone H3K4me2/3 demethylase that is associated with therapeutic resistance in cancer (264). Hinohara et al. (264), demonstrated that inhibition of KDM5B increases sensitivity to endocrine therapy by modulating oestrogen receptor, suggesting the therapeutic potential of this epigenetic demethylating mark. Concerning viral infections, KDM5B was shown to suppress stimulator of interferon genes (STING), a cytosolic DNA sensor that activates downstream transcription factors signal transducer and activator of transcription 6 (STAT6), and interferon regulatory factor (IRF3) through TANK-binding kinase 1 (TBK1) (265). This, in turn, protects the host cells by eliciting an antiviral response and innate immune defence against intracellular pathogens and cancer (265). SARS-CoV proteins were shown to interact with STING and activate the STING-TRAF3-TBK1 complex leading to abnormal alteration and inhibition of type 1 IFN activities that may be associated with severe disease (266). 3C-like (3CL), the main protease and regulator of viral replication for SARS-CoV-2, was shown to inhibit the activation of immune defence machinery by perturbing both RIG-I-like receptors (RLR) and cGAMP binds to stimulator of interferon genes (C-GAS-STING) pathways in human lung cells, suggesting a mechanism that will enable the virus to replicate more efficiently during infection (267). Upregulation of STING and aberrant activities usually correlate with cytokine storm in older people and those who suffer from metabolic disorders (268–271). This may explain the increased COVID-19 severe cases in patients who are older, diabetic, and hypertensive.

## Potential Of Epigenetic Drug Treatment In Covid-19 Infection

Given the above evidence, it is of great interest to determine the impact of various epigenetic marks in COVID-19-related severity and progression for their exploitation for future COVID-19 epigenetic therapy. Although other molecules and pathways (e.g. nuclear factor erythroid 2–related factor 2/Nrf2 and NLRP3) could also be interesting to be mentioned and included in this section (184, 185, 272), we decided to focus on the AHR due to its prominent roles in diverse diseases, including COVID-19. The AHR is a ligand-activating transcription factor that may be activated in response to infection. Its activation has been postulated many times as part of the mechanism behind the cytokine storm and poor clinical outcomes including increased fatalities associated with COVID-19 (145, 178, 179, 273, 274). While cytokines protect against viral infections, they can also be aberrantly regulated and produced excessively. This may unintentionally induce indoleamine 2,3-dioxygenase (IDO), most excessively in male COVID-19 patients, leading to abnormal accumulation of kynurenine that activates the AHR. The AhR is widely expressed in various tissues and thus transcriptionally upregulates the expression of ACE2 receptor in macaques infected with SARS-CoV-2 (273). This enhances SARS-CoV-2 infection resulting in cytopathic effects in various cells and impaired antiviral response, thereby leading to systemic tissue damage and organ failure.

Furthermore, the AHR activation has also been shown to be differentially regulated in comorbidities (e.g. smoking, age, obesity, hypertension, and diabetes) that are strongly linked to poor clinical outcomes of COVID-19 (275–278). Different epigenetic regulation of AHR (181, 181, 279–282) could explain epigenetic regulation of ACE2 receptor, differentially methylated CpG sites observed in COVID-19 and poor clinical outcomes of COVID-19 in some individuals. For instance, activation of the AHR is also associated with reversible hypermethylation in human malignancy including acute lymphoblastic leukaemia (ALL) in *in vitro* studies (181). When demethylated by methylation inhibitor zebularine, AHR-related methylation inhibition restored normal cells phenotype and prevented tumorigenesis (181), suggesting it as a suitable and promising candidate/s for epigenetic therapy.

Likewise, various clinically approved drugs, such as dexamethasone, that are currently used/tested to ameliorate the COVID-19, have been shown to impact the activity of the AHR and to be involved in resistance to therapy, not only in infectious diseases (e.g. tuberculosis) but also in cancer (e.g. melanoma) (279, 280, 283–287). Curcumin and dexamethasone and are 2 classical examples of epigenetics reprogramming drugs and may be helpful to treat COVID-19 toxicity by counteracting the effects of molecules such as the AHR (279, 280). Of note, curcumin can modulate AHR activity (281, 288). Curcumin is a turmeric herb that exerts its potent anti-inflammatory and antioxidant properties by inducing epigenetic reprogramming *via* regulation of DNMTs, HATs, HDACs, and miRNA, reviewed in ref (282).. Various *in vitro* and *in vivo* studies in liver-related diseases have demonstrated that the use of curcumin is associated with suppressed cell growth and reduced liver injury (289, 290). It has been shown that curcumin exerts its activities by inhibiting HDAC activated by the nuclear factor kappa B (NF-κB) pathway (290). It is important to note that this pathway is known to interact with AHR and thus contributing to the regulation of COVID-19-mediated cytokine storm (274). Dexamethasone is a potent anti-oedema/fibrotic corticosteroid agent, and it was shown to accelerate AHR degradation and suppress the expression of its downstream target genes *in vitro* studies (291). Proper dosage of dexamethasone reduced the likelihood of progression of the disease, leading to shorter hospitalisation and reduced fatalities by approximately one third in COVID-19 patients requiring ventilation and by one fifth in those requiring oxygen (280). The use of dexamethasone in cholestatic rats was associated with decreased hepatic inflammation and oxidative stress (292). Investigating epigenetic reprogramming by various receptors and drugs may provide novel therapeutic opportunities to control the current pandemic.

## Future Perspectives And Directions

SARS-CoV-2 may trigger epigenetic alterations affecting the expression of ACE2 and various immunoregulatory genes that play a key role in both immune defence machinery and metabolic pathways on different cells (167, 173–175). This may promote tissue damage and augmenting multi-organ pathology in SARS-CoV-2-infected tissues. Given the evidence above, differentially methylated CpG sites of a wide variety of promoters encoding immunoregulatory genes and ACE2 gene may be the primary COVID-19 epigenetic signature that are set off in response to increased viral infections as part of host immune responses as commonly observed in viral infections. Differential epigenetic regulation associated with ACE2 receptor and AHR (153, 169, 217) may favour viral entry and regulation of ACE2 expression by modulating different epigenetic marks, including DNMTs, H3K27me, KDM5B and SIRT1. These epigenetic marks control metabolic and immunoregulatory pathways, thereby promoting immune evasion and cytokine storm, leading to severe clinical pathologies such as ARDS and widespread tissue damage associated with multi-organ failure (52–61, 175). Detection of epigenetic signatures established in COVID-19 and their dynamics during viral entry and throughout infection (e.g. from asymptomatic to mild symptomatic, severe infection and long persistent symptoms) may be valuable for timely diagnosis and to help designing therapies that may curb the severity of COVID-19 and related fatalities. Type II diabetes mellitus, hypertension and CVD are significant metabolic complications that contribute to the mortality of patients COVID-19. Discovering epigenetic markers linked to these comorbidities and how they impact the severity of COVID-19 may also be valuable for prompting treatment to prevent progression to sequelae that promote COVID-19-associated fatalities mortality.

## Author Contributions

MK conceived the idea and drafted the manuscript. MS, PM-A, IL, GM, TB, PK, HR, PWM, MV, JZ, and HN collected some of the literature and contributed in some sections. MS, PM-A, and SG edited and revised the manuscript. MK and PM-A made final changes, edited and finalised the manuscripts, including the figures. All authors contributed to the article and approved the submitted version.

## Conflict of Interest

The authors declare that the research was conducted in the absence of any commercial or financial relationships that could be construed as a potential conflict of interest.

## Publisher’s Note

All claims expressed in this article are solely those of the authors and do not necessarily represent those of their affiliated organizations, or those of the publisher, the editors and the reviewers. Any product that may be evaluated in this article, or claim that may be made by its manufacturer, is not guaranteed or endorsed by the publisher.
